# Time-To-Treatment of Acute Coronary Syndrome and Unit of First Contact in the ERICO Study

**DOI:** 10.5935/abc.20160138

**Published:** 2016-10

**Authors:** Rafael Caire de Oliveira dos Santos, Alessandra Carvalho Goulart, Alan Loureiro Xavier Kisukuri, Rodrigo Martins Brandão, Debora Sitnik, Henrique Lane Staniak, Marcio Sommer Bittencourt, Paulo Andrade Lotufo, Isabela Martins Bensenor, Itamar de Souza Santos

**Affiliations:** 1 Faculdade de Medicina da Universidade de São Paulo, SP - Brazil; 2 Hospital Universitário da Universidade de São Paulo; Faculdade de Medicina da Universidade de São Paulo, SP - Brazil

**Keywords:** Acute Coronary Syndrome / mortality, Primary Health Care, Aspirin / administration & dosage, Anticoagulants, Time-to-Treatment, Cohort Studies

## Abstract

**Background:**

To the best of our knowledge, there are no studies evaluating the influence of the unit of the first contact on the frequency and time of pharmacological treatment during an acute coronary syndrome (ACS) event.

**Objectives:**

The main objective was to investigate if the unit of first contact influenced the frequency and time of aspirin treatment in the Strategy of Registry of Acute Coronary Syndrome (ERICO) study.

**Methods:**

We analyzed the pharmacological treatment time in 830 ERICO participants - 700 individuals for whom the hospital was the unit of first contact and 130 who initially sought primary care units. We built logistic regression models to study whether the unit of first contact was associated with a treatment time of less than three hours.

**Results:**

Individuals who went to primary care units received the first aspirin dose in those units in 75.6% of the cases. The remaining 24.4% received aspirin at the hospital. Despite this finding, individuals from primary care still had aspirin administered within three hours more frequently than those who went to the hospital (76.8% vs 52.6%; p<0.001 and 100% vs. 70.7%; p=0.001 for non ST-elevation ACS and ST-elevation myocardial infarction, respectively). In adjusted models, individuals coming from primary care were more likely to receive aspirin more quickly (odds ratio: 3.66; 95% confidence interval: 2.06-6.51).

**Conclusions:**

In our setting, individuals from primary care were more likely to receive aspirin earlier. Enhancing the ability of primary care units to provide early treatment and safe transportation may be beneficial in similar settings.

## Introduction

Coronary artery disease (CAD) is the leading cause of mortality^1^ and disability-adjusted life years^2^ worldwide, as well as in Brazil. Previous studies have demonstrated that the quality of pharmacological treatment during an ACS event determines prognosis. The proportion of indicated medications effectively prescribed for a given patient during in-hospital treatment of an ACS event is associated with better in-hospital^3^ and 6-month^4^ survival. Some registry studies, in developing and developed countries, have reported the frequencies of medication prescription in hospitalized ACS patients with heterogeneous results.^5-10^ In Brazil, Nicolau et al.^11^ assessed the use of anti-platelet therapy in 71 hospitals in the Brazilian Registry of Acute Coronary Events (BRACE) study. These authors found that during the first 24 hours of hospitalization, aspirin was administered to 80.2% to 91.2% of patients, and clopidogrel to 42.2% to 67.4% of patients in different regions of the country.

The American Heart Association states that the best option in the case of a suspected ACS event is to activate the mobile emergency medical service (EMS).^12^ This recommendation is based on the fact that this could provide safer transportation and shorter time-to-treatment intervals. However, a substantial proportion of patients seeks medical care directly upon experiencing acute chest pain.^13^ In the Brazilian Health System, individuals who decide to seek medical care directly in this scenario may go to pre-hospital or hospital units, at the patient's discretion. To the best of our knowledge, there are no studies evaluating the influence of the unit of the first contact on the frequency and time to pharmacological treatment during an ACS event.

The Strategy of Registry of Acute Coronary Syndrome (ERICO) study is an ongoing long-term cohort of patients treated for an ACS event in Hospital Universitário da Universidade de São Paulo (HU-USP), a community hospital in the city of São Paulo.^14^ The main objective of the present analysis was to investigate if the unit of first contact influenced the frequency and time to aspirin treatment in the ERICO study. Our secondary objectives were to investigate if the unit of first contact influenced the times to heparin, clopidogrel and thrombolytic treatments.

## Methods

### Study design

The design of the ERICO study has been described in detail elsewhere.^14-17^ Briefly, ERICO is a prospective observational study of 1,085 individuals admitted to a HU-USP because of an ACS event between February 2009 and December 2013. This is a community hospital in Butantã, a neighborhood in São Paulo, Brazil with an area of 12.5 km^2^, an estimated population of 428,000 inhabitants in 2010, and marked socioeconomic inequalities.

Patients can be admitted as transfers from primary care units, by mobile emergency care services, or may seek the emergency department directly. ERICO participants must meet the diagnostic criteria for ST-elevation myocardial infarction (STEMI), non-ST elevation myocardial infarction (NSTEMI) or unstable angina (UA). For a diagnosis of myocardial infarction (MI), both of the following criteria must be present: (I) Symptoms consistent with cardiac ischemia within 24 hours of presenting at the hospital; (II) Troponin I levels above the 99^th^ percentile with a test-specific coefficient of variation <10%. STEMI diagnosis requires both of the following criteria: (I) Criteria for MI diagnosis; (II) One of the following: (a) persistent ST segment elevation of ≥ 1 mm in two contiguous electrocardiographic leads; (b) presence of a new or presumably new left bundle branch block. For NSTEMI diagnosis, participants must present: (I) Criteria for MI diagnosis and (II) Absence of criteria for STEMI diagnosis. For UA diagnosis, all of the following three criteria must be fulfilled: (I) Symptoms consistent with cardiac ischemia 24 hours prior to hospital admission; (II) Absence of MI criteria; (III) At least one of the following: (a) history of coronary artery disease; (b) positive coronary disease stratification test (invasive or noninvasive); (c) transient ST segment changes ≥ 0.5 mm in two contiguous leads, new T-wave inversion of ≥ 1 mm and/or pseudo-normalization of previously inverted T waves; (d) troponin I > 0.4 ng/mL; or (e) diagnostic concordance of two independent physicians. Non-ST elevation acute coronary syndrome (NSTEACS) is a common term that encompasses NSTEMI and UA.

At baseline, trained interviewers obtained data regarding sociodemographic, cardiovascular risk factors and previous medications. During this in-hospital phase, all subjects were treated at discretion of the hospital staff with standard procedures, without any influence from the study protocol. Long-term follow-up is currently ongoing. Participants are contacted annually to update information about their vital status and non-fatal outcomes.

In this ancillary study, we included 830 ERICO participants enrolled from February 2009 to December 2012 who presented to primary care units or directly to the hospital. We reviewed medical charts of the ERICO index event to determine the time-to-treatment and frequency of administration of aspirin, clopidogrel, and heparin for all participants, as well as thrombolytic treatment (streptokinase and tissue plasminogen activators) for participants with a diagnosis of STEMI. From the medical charts, we also retrieved the time of arrival at the unit of first contact and the time of administration of these medications, whether they were administered in primary care facilities or at the hospital. All medications were available at the hospital during the study period. Aspirin was available in all primary care units during the study period, and clopidogrel was available only in one primary care unit, during the last months of the study. Heparin and thrombolytic agents were not available in primary care units.

### Study variables

Time of arrival was defined as the time the patient arrived at the unit of first contact. Time to treatment was defined as the time between patient arrival at the unit of first contact and medication administration. For the main analyses, we categorized time to treatment using a cutoff at 3 hours. Hypertension, diabetes, dyslipidemia, and previous coronary artery disease (CAD) diagnoses were defined by self-report. Smoking status was classified as "never smoked", "past smoker", or "current smoker". Educational level was self-reported and classified as "no formal education", "1 to 7 years of formal education", and "≥ 8 years of formal education".

### Ethical considerations

The study protocol was in accordance with the Declaration of Helsinki. HU-USP review board approved the research protocol (Ethical Committee Approval 866/08). Written informed consent was obtained from all ACS patients admitted to the hospital who agreed to participate in this study, and each subject received a copy of the consent form.

### Statistical analysis

Categorical variables are presented as absolute counts and proportions, and compared using the chi-squared test and Fisher's exact test whenever applicable. Due to the non-normal distribution of age in the sample, we present age (in years) as median and interquartile range, and compare age distributions across groups using the Kruskal-Wallis test. Time to treatment was analyzed for NSTEACS and STEMI participants in separate. We present the bivariate analysis for the association between the unit of first contact and time to treatment using a cutoff at 3 hours. We built crude and adjusted (for age, sex, ACS subtype and educational level) binary logistic regression models to study if the unit of first contact influenced time to treatment. In all models, the dependent variable was the time to treatment, categorized using a cutoff at 3 hours. The Hosmer-Lemeshow test results evidenced an adequate fit for all adjusted models. Significance level was set at 0.05. We used R software version 3.2.0 to conduct these analyses.^18^

## Results

Table 1 shows the baseline characteristics of the study sample. In the group of individuals who presented to primary care units, we observed a higher proportion of men and lower formal educational levels. Both groups were similar according to other characteristics, including age, frequency of hypertension, diabetes, dyslipidemia or previous coronary artery disease.

**Table 1:** Baseline characteristics of the study sample

	Primary care N = 130	Hospital N = 700	p
Age in years; median [Interquartile range]	61.0 [53.3 – 71.0]	62.0 [53.0 – 73.0]	0.72
Male Sex; N (%)	88 (67.7%)	402 (57.4%)	0.037
**Educational level**			
No formal education	24 (18.5%)	84 (12.0%)	0.035
1 to 7 years	61 (46.9%)	299 (42.8%)	
8 or more years	45 (34.6%)	315 (45.1%)	
**ACS subtype**			
STEMI	32 (24.6%)	201 (28.7%)	0.23
NSTEMI	48 (36.9%)	283 (40.4%)	
UA	50 (38.5%)	216 (30.9%)	
Hypertension	100 (77.5%)	530 (77.3%)	1.00
Diabetes	47 (36.7%)	270 (39.7%)	0.59
Dyslipidemia	60 (54.1%)	329 (54.1%)	1.00
Sedentarism	88 (73.3%)	463 (70.8%)	0.65
**Smoking status**			
Never	46 (36.5%)	206 (31.4%)	0.087
Past	35 (27.8%)	250 (38.1%)	
Current	45 (35.7%)	200 (30.5%)	
Previous CAD	34 (28.3%)	187 (29.2%)	0.94

ACS: acute coronary syndrome; STEMI: ST-elevation myocardial infarction; NSTEMI: non ST-elevation myocardial infarction; UA: unstable angina; CAD: coronary artery disease; Missing data: ACS subtype, sex and age have no missing data; Educational level  N = 2 (0.2%);  Hypertension N = 15 (1.8%); Diabetes N = 22 (2.7%); Dyslipidemia N = 111 (13.4%); Sedentarism N = 56 (6.7%); Smoking status N = 48; (5.8%); Previous CAD N = 69 (8.3%).

Complete time-to-treatment information for aspirin, clopidogrel, and heparin was obtained for 746/830 (89.9%) of the study participants. Table 2 shows the proportion of individuals who received aspirin, clopidogrel, or heparin during the index ACS event, according to the unit of first contact. Use of those medications during the index event was almost universal. In addition, we found that 93.6%, 86.1%, and 86.5% of study participants received aspirin, clopidogrel, and heparin during the first 24 hours of hospitalization, respectively. Although aspirin was available in all primary care units during the study period, we found that almost a quarter (24.4%) of study participants who received first contact at primary care did not receive aspirin until arriving at the hospital. Most of those participants who contacted a primary care service and did not receive aspirin had a diagnosis of NSTEACS. Aspirin was administered in the primary care services for 48/69 (69.6%) of the participants with NSTEACS and 20/21 (95.2%) of the participants with STEMI.

**Table 2:** Proportion of 746 individuals with complete time-to-treatment information who received aspirin, clopidogrel, or heparin at any time during the index ACS event, according to the unit of first contact

Medication	Unit of first contact			
	**Primary care (N = 90)**		**Hospital (N = 656)**	p*
	Received at PC unit	**Received at any unit**		
Aspirin	68 (75.6%)	90 (100%)	645 (98.3%)	0.38
Clopidogrel	2 (2.2%)	88 (97.8%)	632 (96.3%)	0.76
Heparin	0	88 (97.8%)	627 (95.6%)	0.57

* We present p-values (Fisher's exact test) comparing the frequency of aspirin, clopidogrel and heparin administration at any time, according to the unit of first contact.

Table 3 shows how many individuals received aspirin, clopidogrel, and heparin up to 3 hours after arrival at the unit of first contact. For individuals with NSTEACS, those who first presented at primary care had higher frequencies of early aspirin administration. Similar results were observed in STEMI patients. We also found significant differences for early heparin administration, with higher frequencies in individuals who first presented at the hospital, in both the NSTEACS and STEMI subsamples.

**Table 3:** Proportion of ERICO participants who received aspirin, clopidogrel, and heparin up to 3 hours after arrival, according to ACS subtype

Non ST-elevation ACS	Primary care N = 69	Hospital N = 468	p*
Aspirin	53 (76.8%)	246 (52.6%)	<0.001
Clopidogrel	15 (21.7%)	152 (32.5%)	0.097
Heparin	8 (11.6%)	125 (26.7%)	0.010
**STEMI**	**Primary care** **N = 21**	**Hospital** **N = 188**	p*
Aspirin	21 (100%)	133 (70.7%)	0.001
Clopidogrel	7 (33.3%)	99 (52.7%)	0.11
Heparin	3 (14.3%)	73 (38.8%)	0.031

* We used chi-square and Fisher's exact tests for comparison. STEMI: ST-elevation myocardial infarction.

Of the 233 participants with STEMI in our sample, we identified 111 who received thrombolytic treatment as the selected reperfusion strategy. We excluded four participants (3.7%) from the primary care group in our analysis of time to thrombolytic treatment because we were unable to retrieve precise information about their time of arrival. There was a non-significant association towards faster time to thrombolytic treatment in those individuals who came directly to the hospital (N=83; 87.4%) compared to those who sought primary care units (N=8; 66.7%; p=0.079 for comparison) using a cutoff at 3 hours. However, the smaller sample size limits the power of this analysis.

Table 4 shows the odds ratios (OR) for the association between faster time from arrival to medical treatment (dependent variable) and first presentation at primary care units (independent variable). The odds ratios for all the covariates in the adjusted models are presented in the Table 5. After adjustment for age, sex, ACS subtype and educational level, we found that initial presentation at primary care units was directly associated with time to treatment with aspirin shorter than 3 hours (p<0.001) but inversely associated with time to treatment with clopidogrel (p=0.013) and heparin (p<0.001) shorter than 3 hours. For the subsample of individuals with STEMI who received thrombolytic therapy, we did not find a significant association between the unit of first contact and time to treatment with thrombolytic therapy shorter than 3 hours in adjusted models (p=0.12). As stated before, the smaller subsample size may also have affected the power of this analysis.

**Table 4:** Odds ratios (95% confidence intervals) for faster time (cutoff set at 3 hours) from arrival to medical treatment

	Crude odds ratios for primary care*	Adjusted odds ratios for primary care*
Aspirin	3.38 (1.93 – 5.93)	3.66 (2.06 – 6.51)
Clopidogrel	0.52 (0.31 – 0.87)	0.52 (0.31 – 0.87)
Heparin	0.32 (0.17 – 0.62)	0.33 (0.17 – 0.63)

* Participants who came directly to the hospital are the reference group. Adjusted models are adjusted for age, sex, acute coronary syndrome subtype and educational level.

**Table 5:** Adjusted odds ratios (95% confidence intervals) for the association between faster time (cutoff set at 3 hours) from arrival to medical treatment in binary logistic models

	Aspirin	Clopidogrel	Heparin
**Unit of first medical contact**			
Hospital	Reference (1.0)	Reference (1.0)	Reference (1.0)
Primary care	3.66 (2.06 - 6.51)	0.52 (0.31 - 0.87)	0.33 (0.17 - 0.63)
**Age**			
By 5-year increase	0.93 (0.88 - 0.99)	0.98 (0.92 - 1.04)	0.99 (0.93 - 1.06)
**Sex**			
Male	Reference (1.0)	Reference (1.0)	Reference (1.0)
Female	1.12 (0.82 - 1.54)	0.95 (0.69 - 1.31)	1.04 (0.74 - 1.45)
**ACS subtype**			
STEMI	Reference (1.0)	Reference (1.0)	Reference (1.0)
NSTEMI	0.39 (0.27 - 0.58)	0.37 (0.25 - 0.53)	0.54 (0.36 - 0.80)
UA	0.55 (0.36 - 0.83)	0.61 (0.41 - 0.89)	0.69 (0.46 - 1.04)
**Educational level**			
No formal education	0.73 (0.44 - 1.21)	0.73 (0.43 - 1.23)	0.72 (0.40 - 1.28)
1 to 7 years	0.99 (0.71 - 1.37)	1.01 (0.73 - 1.41)	1.09 (0.77 - 1.54)
8 or more years	Reference (1.0)	Reference (1.0)	Reference (1.0)

ACS: acute coronary syndrome; STEMI: ST-elevation myocardial infarction; NSTEMI: non ST-elevation myocardial infarction; UA: unstable angina.

## Discussion

We found that 24.4% of study participants who first presented at primary care units did not receive aspirin before being transported to the hospital. Nevertheless, these individuals were more likely to receive early aspirin treatment compared to participants who came directly to the hospital. Heparin and clopidogrel were administered earlier in those who came directly to the hospital. There was a non-significant association pointing towards faster treatment with thrombolytic therapy in STEMI patients that came directly to the hospital, when this was the selected strategy for reperfusion.

We describe similar or higher frequencies of aspirin, clopidogrel, and heparin use during the acute phase of ACS treatment in our sample compared to those found in other studies. The frequencies of use of aspirin, clopidogrel, and heparin at any time during the in-hospital phase of ACS treatment were 98.5%, 96.5%, and 95.8% respectively in our study. When limited to the frequencies of prescription during the first 24 hours of hospitalization, the use of aspirin, clopidogrel, and heparin in ERICO were 93.6%, 86.1%, and 86.5% in our sample. Bajaj et al.^7^ analyzed data from 16,618 patients in Canada and found that 92% received aspirin, 63% clopidogrel, and 88% heparin during the first 24 hours of hospitalization.

Some studies evaluated the frequency of medication administration during an ACS event in developing countries as well. In 65 hospitals located in six Middle Eastern countries, Shehab et al.^6^ analyzed data from 7,930 men and women and described that 98.4% of men and 98.2% of women received in-hospital aspirin and 79.2% of men and 64.9% of women received clopidogrel. In the ACCESS prospective observational study^8^ the authors analyzed 12,068 adults hospitalized with a diagnosis of ACS at 134 sites in 19 countries in Africa, Latin America, and the Middle East. The authors found that 93% of the patients received aspirin and 81% received clopidogrel (or other thienopyridines). The Kerala ACS Registry,^9^ in India, collected data on 25 748 patients and describe frequencies of medication administration of 93.0%, 95.1% and 70.0% for aspirin, clopidogrel and any heparin, respectively. Bandara et al.^10^ analyzed in Sri Lanka 81 patients with STEMI and, in that study, 99% of study participants received aspirin and 97% clopidogrel. Compared to ERICO, studies in developing countries showed similar frequencies for aspirin administration, but lower frequencies for heparin and sometimes clopidogrel use. Although it is not possible to draw definite conclusions, inequalities in study populations and local protocols may be partially responsible for these differences.

In Brazil, the aforementioned BRACE study^11^ found that aspirin and clopidogrel were prescribed at rates of 89.0% and 59.7%, respectively, during the first 24 hours of hospitalization. Specifically, an analysis of a subgroup of hospitals in the Southeast region of the country (where HU-USP is located) in the BRACE study, found aspirin and clopidogrel administration frequencies during the first 24 hours of hospitalization of 87.0% and 67.4%, respectively. We may raise some hypotheses to explain the differences between these two studies. Multicenter registry studies tend to over-represent tertiary centers. Individuals in tertiary centers may have easier access to early angioplasty. This may have postponed the administration of clopidogrel in some study participants,^19^ to ensure that surgical planning (if needed) would not be impaired. In addition, individuals who seek care in tertiary centers generally have more comorbidities than patients in community hospitals. We may speculate that registries in tertiary centers may include a higher proportion of individuals with contraindications to medical treatment, and this may partially explain the higher medical treatment frequencies found in ERICO.

A substantial part of ACS mortality occurs in the first hours of symptoms.^20^ In addition, prompt diagnosis and adequate treatment influence the occurrence of long-term fatal^21^ and non-fatal^22^ complications. Timely prescription may also be a marker of adequate monitoring and agile decision-making in the emergency department. In this sense, exploring bottlenecks in early medication use is important, and may reveal alternative options in the system of care for ACS patients. Most evidence of factors influencing time-to-treatment is based on information about aspirin administration^23^ and, in patients with STEMI, thrombolytic treatment^24^ or revascularization procedures.^25^ In our study, a quarter of ACS patients who sought care at primary care units did not receive aspirin before being transferred to the hospital, although aspirin was a widely available medication in all primary care units during the study period.^26^ The percentage of failure in aspirin administration in the primary care units was higher in NSTEACS patients than in participants with STEMI. One possible explanation is that it is more difficult to recognize and diagnose NSTEACS patients, in whom electrocardiographic changes may be absent or non-specific. However, aspirin administration is indicated upon suspicion of ACS in patients without contraindications, and does not require diagnostic confirmation. Improving recognition and early treatment with aspirin in primary care units, along with safe transportation, may be beneficial to patients. The potential of such a strategy is evident, because even considering that some of the ACS patients who sought care at primary care units only received aspirin at the hospital, the frequency of individuals with early aspirin administration was higher in the primary care group. This consequently indicates a potential window for improvement, because we may speculate that specific interventions targeting prompt administration of aspirin in these units could enhance these results. As for clopidogrel and heparin, these medications are typically available only in hospitals, in our study setting, and only during the last months of the study period did a single primary care unit have access to clopidogrel. We found higher odds of receiving clopidogrel and heparin up to 3 hours after arrival in individuals who came directly to the hospital. However, unlike aspirin and reperfusion therapies, it is not possible to establish a direct benefit of this earlier treatment, nor to determine if enhancing the availability of clopidogrel and heparin in primary care units would be useful and/or cost-effective. Finally, we found a non-significant association pointing towards faster thrombolytic treatment in STEMI patients who came directly to the hospital. Pre-hospital thrombolysis is a potential measure for reducing time-to-treatment with these agents,^27,28^ but it is not yet a widespread reality in our setting. Although our data do not permit definite conclusions, we may speculate that the availability of pre-hospital thrombolysis could reduce the time-to-treatment with thrombolytic therapy in STEMI individuals who sought care at PC units.

Our study has some strength. It focuses on ACS treatment in community hospitals, a common scenario^29^ but one which is frequently under-represented in ACS registries and/or post-ACS cohorts. In fact, some findings in our study, such as the frequencies for early clopidogrel prescription, are at least partially explained by differences between community and tertiary care settings, highlighting the importance of this present article. We were able to retrieve time-to-treatment information from most of the sample. This included not only frequencies of medication administration during the first day of admission, which most studies present, but also detailed information about the first hours of treatment. These data may better reflect differences arising from the unit of first contact. The study also has some limitations. It is a single-center study based on a community hospital in Brazil's largest city and its associated primary care units. We must emphasize that it is not possible to extend our results to all settings, and our conclusions may be suitable only in settings similar to the present study. In specific, in the city of São Paulo, some primary care units (as those in the present study) include a service called *Atendimento Médico Ambulatorial* (AMA - Medical Outpatient Consultation, in Portuguese), aimed to provide easier access to individuals with acute symptoms. We did not include information about contraindications in the present study. However, the frequencies of aspirin, clopidogrel and heparin prescriptions during the in-hospital treatment period were close to 100%, and it is unlikely that our results would be different if information about contraindications were available. In addition, as also studied by other authors,^5-11^ our focus was mainly on the time elapsed between contact and treatment, and we did not assess medication doses or discontinuation. Our objective in the present study was to evaluate if the unit of first contact influenced the frequency and time to pharmacological treatment. Therefore, it is not possible to infer and quantify, directly from the study results, the impact of time to pharmacological treatment on major cardiovascular events. However, main management guidelines^30,31^ highlight the importance of prompt recognition and treatment as a useful strategy to reduce the mortality risk present in the first hours of an untreated ACS event. In this scenario, time to treatment may be a useful marker of the quality of care during an ACS event.

## Conclusions

Study participants who first presented at primary care units were more likely to receive early aspirin treatment compared to participants who came directly to the hospital. However, almost a quarter of study participants who first presented at primary care units did not receive aspirin before being transported to the hospital. In our setting, enhancing the ability of primary care units to provide early treatment (which may include protocols and staff training to guarantee early aspirin use in patients who do not present contraindications) and safe transportation may be beneficial.
